# Effects of *Cimicifuga racemosa* extract Ze450 on mitochondria in models of oxidative stress in neuronal cells

**DOI:** 10.1016/j.dib.2018.10.092

**Published:** 2018-10-26

**Authors:** Malena Rabenau, Matthias Unger, Jürgen Drewe, Carsten Culmsee

**Affiliations:** a Institut für Pharmakologie und Klinische Pharmazie, Biochemisch-Pharmakologisches Centrum Marburg, Philipps-Universität Marburg, Karl-von-Frisch-Straße 1, 35032 Marburg, Germany; b Preclinical Research, Max Zeller Soehne AG, Romanshorn, Switzerland; c Center for Mind Brain and Behavior, 35032 Marburg, Germany

## Abstract

This data article describes the influence of *Cimicifuga racemosa* extract Ze 450 on neuronal cells in models of glutamate-induced excitotoxicity and cell death induced by oxidative stress. Effects of Ze 450 on glutamate-mediated excitotoxicity were assessed in primary cortical rat and mouse neurons and, further, glutamate-mediated oxidative stress was analyzed in HT22 cells lacking ionotropic glutamate receptors. This study especially focusses on mitochondrial parameters like mitochondrial ROS formation, mitochondrial membrane potential, ATP production and mitochondrial integrity. Further the effects of Ze 450 on lipid-peroxidation, metabolic activity, cell proliferation and cell death were assessed under control conditions and oxidative challenge evoked by millimolar concentrations of glutamate in HT22 cells. These data support the findings in HT22, mHypo and HepG2 liver cells (Rabenau et al., 2018) [1].

**Specifications table**

**Table:** 

Subject area	*Pharmacology*
More specific subject area	*Phytopharmacology, Neuroscience*
Type of data	*Graph*
How data were acquired	*Absorbance and luminescence measurements*
	*Fluorescence microscopy*
	*FACS*
Data format	*analyzed*
Experimental factors	*Cimicifuga racemosa extract Ze 450 was produced by a standardized procedure was exposed to HT22 cells and primary cortical rat and mouse neurons.*
Experimental features	*HT22 cells were challenged with glutamate to induce oxidative damage. Cell death was analyzed using Annexin V/PI FACS staining, metabolic activity was determined via MTT assay, cell proliferation was measured with the xCELLigence system, mitochondrial integrity was analyzed via MitoTRACKER red staining and following fluorescence microscopy and moreover lipid-peroxidation (BODIPY), mitochondrial membrane potential (TMRE) and mitochondrial ROS formation (MitoSOX) were determined via FACS analysis.*
	*Primary cortical rat and mouse neurons were exposed to glutamate and co-treated with Ze 450 to determine metabolic activity (MTT assay).*
Data source location	*Marburg, Germany*
Data accessibility	*Data are within the article.*
Related research article	*Metabolic switch induced by Cimicifuga racemosa extract prevents mitochondrial damage and oxidative cell death,*DOI:10.1016

**Value of the data**

• These data present the protection of neuronal cells in models of oxidative stress by Ze 450.
• These data indicate that Ze 450 is involved in preventing oxidative damage in neurons, and could be further investigated as a pharmacologic agent for diseases related with increased ROS formation.
• Ze 450 protects mitochondria from oxidative damage and offers new approaches for studies on its mechanism of action.

## 1 Data

**Fig. 1:**
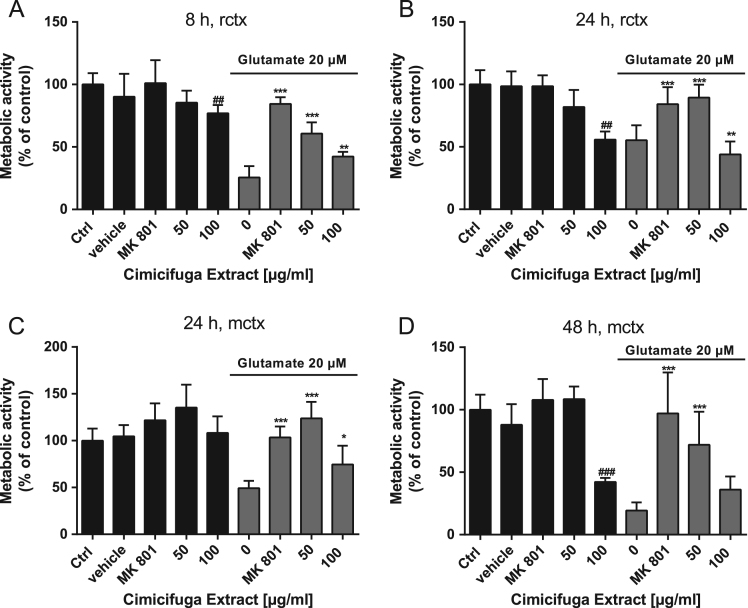
Ze 450 protects against excitotoxicity in primary cortical rat and mouse neurons. (A+B) MTT assay demonstrated protection with concentrations greater than 50 μg/ml of Ze 450 against glutamate-mediated (20 μM, 8 h or 24 h) reduction of metabolic activity (*n* = 6) in primary cortical rat neurons. (C+D) MTT assay demonstrated protection concentrations greater than 50 μg/ml of Ze 450 against glutamate-mediated (20 μM, 24 h or 48 h) reduction of metabolic activity (*n* = 6) in primary cortical mouse neurons. All data are given as mean+SD ##*p* < 0.01, ###*p* < 0.001 compared to untreated control; *p < 0.05, **p < 0.01, ***p < 0.001 compared to glutamate treated primary cortical cells (ANOVA Scheffé׳s test).

The dataset of this data article provides the information on the effects of Ze 450, a *Cimicifuga racemosa* extract that was produced by a standardized procedure in models of oxidative stress in HT22 neuronal cells [2]. Further, we analyzed protective effects of Ze450 in primary cortical mouse and rat neurons exposed to glutamate (Fig. 1).

**Fig. 2:**
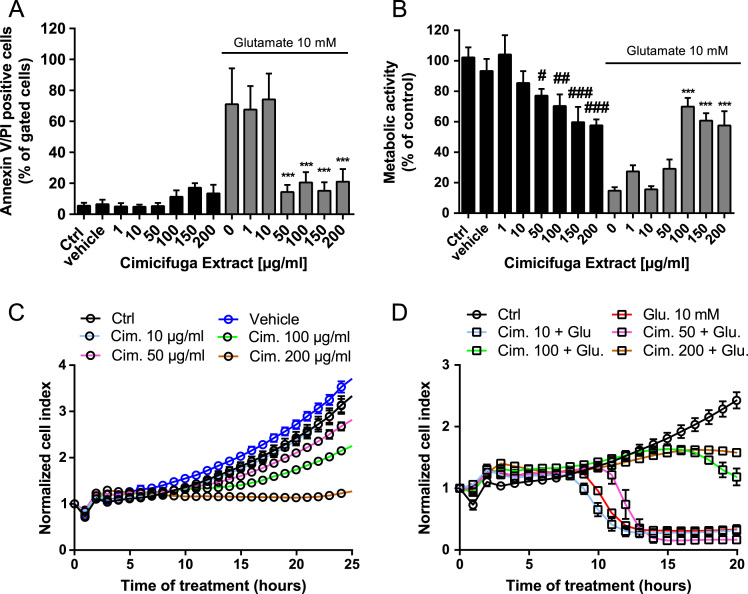
Ze 450 prevents against glutamate-mediated cytotoxicity and reduction in proliferation. (A) Annexin V/PI staining and subsequent FACS analysis showed reduced cell death in HT22 cells treated with concentrations greater than 50 μg/ml Ze 450 and glutamate compared to glutamate treated HT22 cells (10 mM, 16 h). (B) MTT assay revealed protection by Ze 450 at concentrations greater than 100 μg/ml of Ze450 against glutamate-mediated (10 mM, 16 h) reduction of metabolic activity in HT22 cells (*n* = 6/treatment condition). (C+D) Real-time impedance measurements showed reduced proliferation with concentrations greater than 100 μg/ml of Ze 450 and prevented glutamate-mediated reduction of cell index. Data were obtained in the same experiment but showed separately for better visualization. All data are given as mean+ or ±SD #*p* < 0.05, ##*p* < 0.01, ###*p* < 0.001 compared to untreated control; ***p < 0.001 compared to glutamate treated HT22 cells (ANOVA Scheffé׳s test).

**Fig. 3:**
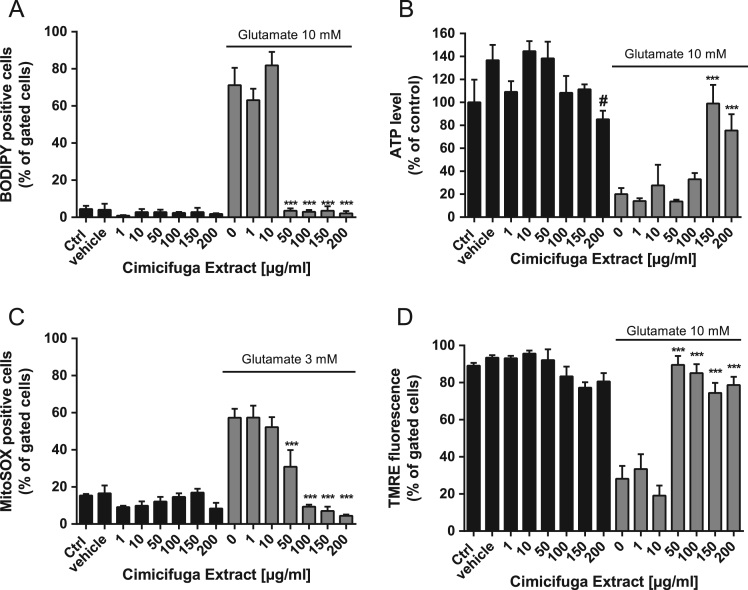
Ze 450 mediates protection against glutamate-induced mitochondrial impairment. (A) HT22 cells were stained with BODIPY 581/591 and changes of fluorescence were detected by FACS analysis after 16 h Ze 450 and glutamate (10 mM) co-treatment. Concentrations greater than 50 μg/ml of Ze 450 prevented the formation of lipid peroxides compared to HT22 control cells (*n* = 3/treatment condition). (B) ATP assay. Concentrations greater than 200 μg/ml reduced ATP level, while 150 μg/ml of Ze 450 mediated protection against glutamate-induced ATP depletion (10 mM, 16 h, *n* = 6/treatment condition). (C) MitoSOX staining and subsequent FACS analysis. Concentrations greater than 50 μg/ml mediated protection against glutamate-mediated (10 mM, 16 h) mitochondrial ROS formation (*n* = 3/treatment condition). (D) TMRE staining and following FACS analysis. Concentrations greater than 50 μg/ml mediated protection against glutamate-mediated (10 mM, 16 h) loss of mitochondrial membrane potential (*n* = 3/treatment condition). All data are given as mean +SD #*p* < 0.05 compared to untreated control; ***p < 0.001 compared to glutamate treated HT22 cells (ANOVA Scheffé׳s test).

**Fig. 4:**
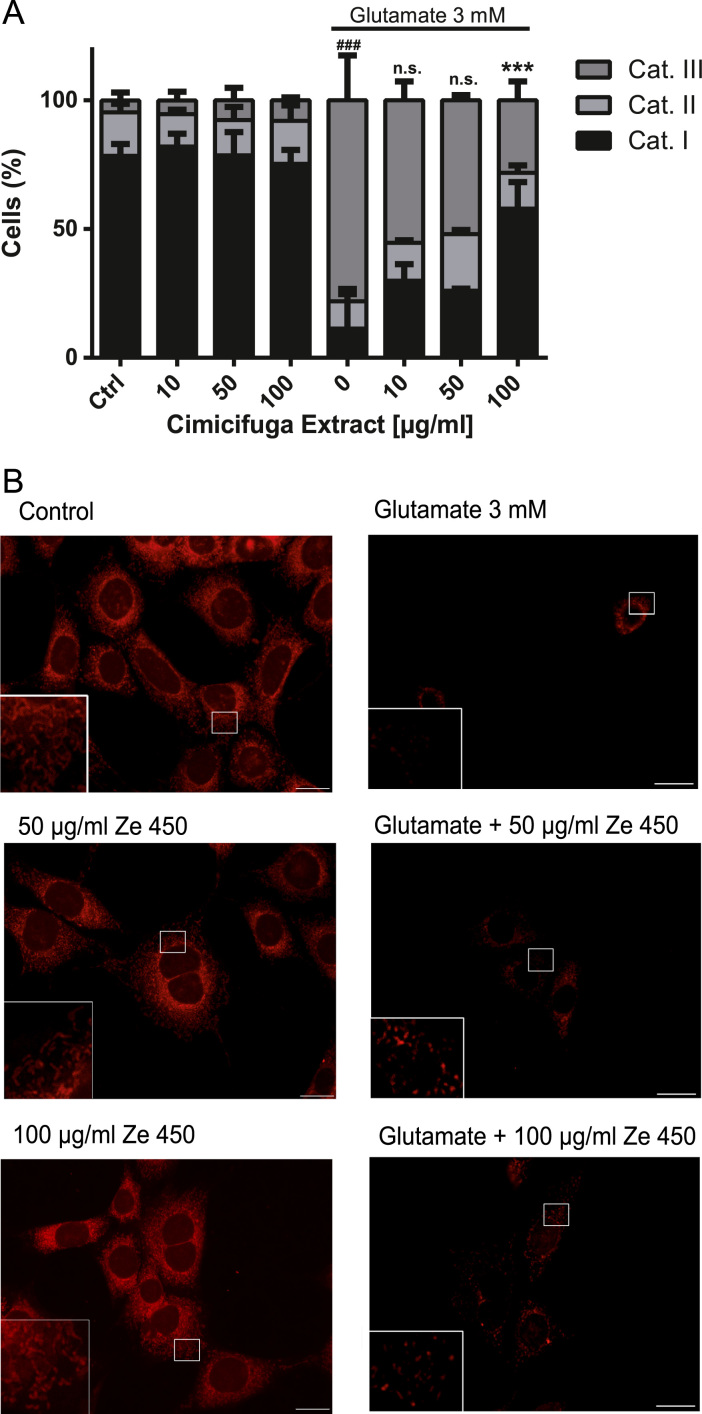
Mitochondrial morphology. (A) Quantification of mitochondrial morphology in HT22 cells exposed to Ze 450 and glutamate (3 mM, 16 h). Glutamate exposure led to an increase in category III mitochondria, which was prevented by concentrations greater than 100 μg/ml of Ze 450. Mean values were pooled from three independent experiments, where mitochondrial morphology was determined from at least 500 cells per condition without knowledge of treatment history. (B) Epifluorescence images were acquired with a fluorescence microscope (DMI6000, Leica, Germany) using 63 × 1.4 NA oil immersion objective (scale bar 20 μm).

With respect to analyzing mitochondrial parameters and oxidative cell toxicity, we used a well-established *in vitro* model of glutamate-induced oxidative stress [3]. This dataset shows the effects of Ze 450 on cell death (Annexin V/PI), metabolic activity (MTT) and cell proliferation (xCELLigence) on HT22 cells under control conditions and with glutamate challenge (Fig. 2). Mitochondrial parameters were analyzed using ATP assay, BODIPY 581/589, MitoSOX and TMRE FACS analysis (Fig. 3). Mitochondrial integrity was evaluated after staining with MitoTracker Red and quantification of fluorescence imaging (Fig. 4).

## 2 Experimental design, materials, and methods

### 2.1 Cell culture

HT22 cells (kindly provided by David Schubert, Cellular Neurobiology Laboratory, Salk Institute for Biological Studies, La Jolla, California, USA) were grown in Dulbecco׳s modified Eagle medium (DMEM, Capricorn Scientific GmbH, Germany) supplemented with 10% heat-inactivated fetal calf serum (Merck, KGaA, Germany), 100 U/ml penicillin, 100 mg/ml streptomycin (Merck KGaA, Germany)and 2 mM glutamine (Merck KGaA, Germany). To induce cell death, 3–10 mM glutamate (Merck KGaA, Germany) was added to the medium for the indicated amount of time (8–16 h).

Primary cortical neurons were prepared from embryonic mouse brains (E18) as described [4]. Dissociated neurons were seeded with 50,000 cells per well onto polyethyleneimine (PEI) coated 96 well plates. Cultures were grown in neurobasal medium (ThermoFisher Scientific, Germany) supplemented with 1.2 mM glutamine, 2% B27 supplement (Invitrogen, USA), 100 U/ml penicillin and 100 mg/ml streptomycin. Treatment with Ze 450 and glutamate (20 μM) was carried out on day ten in culture. MK-801 (Merck KGaA, Germany) was added at a concentration of 10 μM.

Primary cortical rat neurons (kindly provided by Prof. Schratt, Marburg University, Germany) were plated on PEI coated 96 well plates with 20,000 cells/well and handled similarly to the mouse neurons.

### 2.2 Cimicifuga racemosa extract Ze 450

The ethanolic (v/v) *C. racemosa* dry extract Ze 450 was manufactured of dried roots and rhizomes and obtained from Max Zeller and Soehne AG (Romanshorn, Switzerland). The content of triterpene glycosides was 6.4%. Ze 450 was dissolved in 60% ethanol (v/v) (Carl Roth GmbH, Germany) for all experiments. Ze 450 conforms to the herbal preparation B, which was granted well-established use in the 2010 Community herbal monograph on *C. racemosa* by the HMPC [5]. HPLC fingerprints of two extract batches of Ze 450 have been recently published [6].

### 2.3 Cell viability

Cell proliferation was analyzed in real time by measuring electrical impedance as described before [7]. Metabolic activity as an indicator for cell viability was quantified using the MTT assay [3]. Viable and metabolically active cells convert 3-(4,5-dimethylthiazol-2-yl)-2,5-diphenyltetrazolium bromide (MTT, Merck KGaA, Germany), which was added at a concentration of 2.5–5 mg/ml for 1 h at 37 °C to the culture medium, into purple formazan. Absorbance was measured at 570 nm versus 630 nm with FluoStar (BMG Labtech, Germany) after dissolving in DMSO (Carl Roth GmbH, Germany).

Cell death of HT22 cells treated with Ze 450±glutamate was detected using the Annexin-V-FITC/PI Detection Kit (PromoCell, Germany) followed by fluorescence-activated cell sorting (FACS, guava easyCyte, Merck KGaA, Germany). Annexin-V-FITC was excited at 488 nm, emission was detected through a 525 ± 30 nm bandpass filter. Propidium iodide was excited at 488 nm, fluorescence emission was detected using a 690 ± 50 nm bandpass filter. Data were collected from at least 5000 cells with at least three replicates per condition.

### 2.4 Lipid peroxidation

After 7 h of treatment time HT22 cells were stained with BODIPY 581/591 C_11_ (Invitrogen, USA) for 1 h (37 °C, 4.5% CO_2_) and harvested for FACS analysis. Lipid peroxidation was analyzed by recording green (emission: 525 nm/30) and red (emission: 585 nm/50) fluorescence with 488 nm excitation wavelength of at least 5000 cells of at least three replicates per condition. Levels of lipid peroxidation were calculated by the analysis of the fluorescence shift from green to red fluorescence.

### 2.5 Mitochondrial ROS formation

MitoSOX red (Invitrogen, USA) is selectively targeted to the mitochondria, where it is oxidized by superoxides exhibiting red fluorescence. For detection of mitochondrial ROS formation, MitoSOX red was applied for 30 min at 37 °C and cells were harvested for FACS analysis [2]. Increasing red fluorescence correlating with the formation of mitochondrial ROS was detected by FACS analysis (excitation 488 nm, emission 690/50 nm). Data were collected from at least 5000 cells and three replicates per condition.

### 2.6 Mitochondrial morphology

MitoTracker® Deep Red FM (Invitrogen, USA, 200 nM) was used to visualize changes in mitochondrial morphology. HT22 cells were seeded in 8-well ibidi slides (Ibidi GmbH, Germany) with 14,000 cells per well and treated with Ze 450 and glutamate. After 16 h of treatment time, MitoTracker® Deep Red was added to cells and incubated for 30 min at 37 °C. Cells were fixed with 4% paraformaldehyde for 20 min at room temperature (RT). Mitochondrial morphology was analyzed by categorizing cells as previously described [8]. Category I mitochondria were organized in a tubular network, category II mitochondria were fragmented but equally distributed throughout the cytosol, whereas category III mitochondria were fully fragmented and surrounding the nucleus. At least 500 cells per condition were counted without knowledge of treatment history. Images were acquired using a Leica DMI6000 epi-fluorescence microscope (63× objective), using an excitation wavelength of 620 nm (bandpass filter) and detecting emissions using a 670 nm long pass filter.

### 2.7 ATP measurements

ATP levels were detected using the ViaLight^™^plus Kit (Lonza, USA) according to manufactures protocol. Twenty-four hours post seeding in 96-well plates (6000 cells per well), cells were treated with Ze 450 and glutamate. After 8 h treatment cells were transferred into a white 96 well plate and ATP levels were analyzed by luminescence detection with FluoStar OPTIMA (BMG Labtech, Germany).

### 2.8 Mitochondrial membrane potential

After treatment with Ze 450 and glutamate, HT22 cells were stained with TMRE (0.4 nM for 30 min at 37 °C, MitoPT ΔΨm Kit, Immunochemistry Technologies, USA) and harvested for TMRE fluorescence measurement *via* FACS analysis [2]. Upon loss of the mitochondrial membrane integrity and, thus, membrane potential, a loss of TMRE fluorescence can be detected by FACS analysis (excitation 488 nm, emission 690/50 nm). Data were collected from at least 5000 cells and three wells per condition.

### 2.9 Statistical analysis

All data are given as mean+ or ±standard deviation (SD). Statistical comparison between treatment groups was performed by analysis of variance (two-way ANOVA) followed by Scheffé’s post hoc test and a *p*-value < 0.05 was considered to be statistically significant. Calculations were executed with Winstat standard statistical software (R. Fitch Software, Germany) and visualized using GraphPad Prism software (GraphPad Software, United States).
