# Supplementary material for: Effects of Cimicifuga racemosa extract Ze450 on mitochondria in models of oxidative stress in neuronal cells
Source: Data Brief. 2018 Oct 26;21:1872–9. doi: 10.1016/j.dib.2018.10.092 (PMC6260237; doi:10.1016/j.dib.2018.10.092)
Supplement: Supplementary file 1 — Supplementary material [file mmc1.pdf]

AUTHOR DECLARATION

We wish to draw the attention of the Editor to the following facts which may be considered as potential conflicts of interest and to significant financial contributions to this work. Matthias Unger and Jürgen Drewe are employees of the Max Zeller Soehne AG, the study was sponsored by funding of the Max Zeller Soehne AG to TransMIT Project Division for "Mitochondrial Mechanisms of Neuronal Processes" of Prof. Dr. Carsten Culmsee

We confirm that the manuscript has been read and approved by all named authors and that there are no other persons who satisfied the criteria for authorship but are not listed. We further confirm that the order of authors listed in the manuscript has been approved by all of us.
We confirm that we have given due consideration to the protection of intellectual property associated with this work and that there are no impediments to publication, including the timing of publication, with respect to intellectual property. In so doing we confirm that we have followed the regulations of our institutions concerning intellectual property.

We understand that the Corresponding Author is the sole contact for the Editorial process (including Editorial Manager and direct communications with the office). He is responsible for communicating with the other authors about progress, submissions of revisions and final approval of proofs.

We confirm that we have provided a current, correct email address which is accessible by the Corresponding Author and which has been configured to accept email from (culmsee@staff.uni-marburg.de)

Signed by all authors as follows:

Malena Rabenau Malena Rabenau (Marburg, 21.02.18)

Matthias Unger Matthias Unger (22.02.2018)

Jürgen Drewe Jürgen Drewe 23. Feb. 2018

Carsten Culmsee Carsten Culmsee (Marburg, 21.2.2018)

Prof. Dr. Carsten Culmsee
Institut für Pharmakologie und Klinische Pharmazie, FB 16
Philipps-Universität Marburg
Karl-von-Frisch-Str. 1
D-35032 Marburg, Germany
