# Supplementary material for: Effects of Cimicifuga racemosa extract Ze450 on mitochondria in models of oxidative stress in neuronal cells
Source: Data Brief. 2018 Oct 26;21:1872–9. doi: 10.1016/j.dib.2018.10.092 (PMC6260237; doi:10.1016/j.dib.2018.10.092)
Supplement: Supplementary file 2 — Supplementary material [file mmc2.docx]

**Conflict of interest**

Matthias Unger and Jürgen Drewe are employees of the Max Zeller Soehne AG.
